# Impact of daily *supplementation of Spirulina platensis* on the immune system of naïve HIV-1 patients in Cameroon: a 12-months single blind, randomized, multicenter trial

**DOI:** 10.1186/s12937-015-0058-4

**Published:** 2015-07-21

**Authors:** Marthe-Elise Ngo-Matip, Constant Anatole Pieme, Marcel Azabji-Kenfack, Bruno Moukette Moukette, Emmanuel Korosky, Philippe Stefanini, Jeanne Yonkeu Ngogang, Carl Moses Mbofung

**Affiliations:** 1 National Institute of Agro-Industrial Sciences, University of Ngaoundere, POBOX 455, Ngaoundere, Cameroun; 2 Department of Physiological Sciences and Biochemistry, Faculty of Medicine and Biomedical Sciences, University of Yaounde 1, POBOX 1634, Yaounde, Cameroon; 3 Spirulina Producer’s Federation of France, Hyères, France; 4 Ecole de formation Agricole de Hyères, Hyères, France

**Keywords:** *Spirulina platensis*, HIV-naive, CD4 cells, *Balance diet*, Viral load, Clinical trial

## Abstract

**Background:**

Micronutrient deficiencies occur early in Human Immunodeficiency Virus (HIV) infections they have reverse effects on the nutritional status. The diet supplementation with a natural nutraceutical rich in proteins and micronutrient like *Spirulina platensis*, may be effective and efficient in delaying HIV disease progression by frequently reported improvement in immune response.

**Methods:**

A prospective single-blind, randomized, multicenter study conducted on 320 HIV-1 ARV-naïve participants for 12 months. Participants received either *S. platensis* supplementation and standard care or standard care and local balanced diet without *S. platenis.* Selected hematological and biochemical as well as CD4 count cells, viral load copies were assessed at three separate times.

**Results:**

Among the 169 ART-naïve participants enrolled in the study, the female was mostly represented (67.1 %). The significant increase of CD4 count cells (596.32–614.92 cells count) and significant decrease of viral load levels (74.7 × 10^3^–30.87 × 10^3^ copies/mL) of the patients who received a supplementation of *S. platensis* was found after 6 months of treatment. Haemoglobin level was also significantly higher in the same group while the fasting blood glucose concentration decreased after 12 months compared to control.

**Conclusion:**

A daily supplementation with *S. platensis* to diet combined with a reasonable balanced diet has significantly increased the CD4 cells and reduced the viral load after 6 months. Further studies are recommended among a large specific group of people infected by the HIV in order to investigate the mechanisms involved on the effect of *S. platensis* on immune system.

## Introduction

One of the several Public Health problems faced in Sub-Saharan Africa is HIV/AIDS. In Cameroon, about 4.3 % of the population are infected and this prevalence is particularly higher among women and individuals aged between 15 and 49 years [1, 2]. In Africa in general and Cameroon in particular, HIV/AIDS pandemic has created a new form of vulnerability with regards to food security and nutrition. Poverty and food insecurity impacts feeding habits and the reduce access to a nutrition rich in macro and micronutrients which lead to the health impairment mainly in HIV patients [3].

Malnutrition potentialises the effects of HIV infection on the individual by promoting chronic fatigue and disease progression, the latter lead to the morbidity and earlier death [4]. Malnutrition is an important factor that affects the care of patients infected by the HIV, their treatment and it contributes to the development of opportunistic infections such as tuberculosis [5, 6]. Malnutrition and HIV also have similar deleterious effects on the immune system and in both cases there are reduced CD4 and CD8 T-lymphocyte levels and impaired serological response after immunizations [7]. The synergistic effects of malnutrition and HIV on the immune system occur in a vicious cycle in which the decrease of immunity associated with higher risk of development of other infectious diseases. HIV-infected adults and children are encouraged to consume healthy diets and individuals with HIV/AIDS require greater protein and micronutrient intake to support a weakened immune system [8]. International organizations have called for food assistance to be integrated into HIV treatment and prevention programs, but evidence-based guidance on how exactly to implement such programs, on what beneficiaries to target, and on what the optimal components or duration of food assistance should be not yet well described [9]. Therefore, dietary supplements are often used by people living with HIV infection to ‘boost immune functioning’ such as mega-dose vitamins, anti-oxidants and body cleansing products such as teas and herbs to remove ‘toxins’ [10].

To fight against nutrients deficiency mainly to HIV patients, the World Health Organization (WHO) recommends nutrients intake of each required micronutrients, which may be taken through nutrient supplementation [6, 11]. Due to the difficulties to access to a balanced diet, *Spirulina platensis* a cyanobacterium available in sub-Saharan Africa has been used as food source. *S. platensis* represents an important staple diet in humans and has been used as a source of protein and vitamin supplement in humans without any significant side-effects [12]. It contains protein (up to 70 %), vitamins, especially B12 and provitamin A (β-carotenes), and minerals, especially iron and other bioactives moleucles such as phenolic acids, tocopherols and glinolenic acid [2, 12]. Several studies demonstrated beneficial effects of *S. platensis in vitro* and *in vivo* as well as its therapeutic functions [13, 14]. In addition, some studies show that spirulina has beneficial effects on the treatment of malnutrition and on other pathologies such as obesity, hypercholesterolaemia, arterial hypertension and diabetes mellitus [15–17]. This study was performed, to assess the potential effects of 10 grams of daily supplementation of *S. platensis* with a local balance diet on the level of CD4 cells count and viral load during six months by treatment naïve HIV–infected persons.

## Methods

### Selection of population of study

We carried out a longitudinal study in a randomized cohort from January 2011 to February 2012. Patients aged between 18–65 years, HIV–infected naïve to antiretroviral treatment and with CD4 count ≥400 cells/μL were eligible. Patients who had their CD4 below 400 cells/μL during the follow-up were excluded. Informed consent was obtained from every subject prior to the participation for the study. The study was approved by the Cameroon National Ethic Committee under the following reference number: 123/CNE/SE/2011, Cameroon. The subjects were recruited through the medical file obtained from the Day Care Clinic of Central Hospital of Yaounde (CHY) and of the Biyem-Assi District Hospital. Investigation and intervention were carried out at the Etoug-Ebe Health Center.

### Selection, randomization, treatment allocation and follow up

**Fig. 1:**
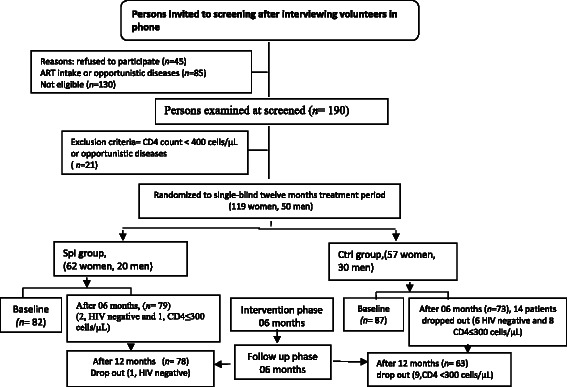
Flow chart describing progress of participants through the Spirulina platensis supplementation trial

Selected participants (169 subjects) were included in the study. They were divided into two groups: 87 subjects to the control group (first group) and 82 subjects to the intervention group (second group). HIV infected patients were selected after checking patients files for CD4 counts over 400 cells/μL who were ARV naïve to treatment (Fig. 1). The two groups were matched with respect to age, sex, and CD4 cells counts. At the inclusion in the study, patients had been fasting for at least eight hours overnight. The patients of the first group were advised who to take a local balance diet while patients of the second group were asked to add 10 g of *S. platensis* to their usual diet during the first six months. The *S. platensis* was given in package of powder doses of 10 grams per day. The next six months were for the follow-up without *S. platensis* powder. Subjects were treated “as usual care” treatment in Cameroon. To monitor the biological parameters a standardized questionnaire for demographic characteristics, clinical, quality of live was filled by the subjects at the baseline, during and at the end of the trial.

### Determination of biochemical parameters

The biochemical indexes were monitored three times: at the baseline, after six and twelve months where the blood samples were collected in three tubes of 4 mL. The first tube was used to determine the CD4 lymphocytes count by all patients. A volume of 50 μL of peripheral blood collected was labeled for Flow Cytometry using BD CD4 multitest kits according to Manufacturer’s instructions. Samples were the acquired using BD FACS CALIBUR II Machine (BD Biosciences, Germany). CD4 T cells values were expressed as cells/μL. The second tube was used for the determination of the viral load with an Abbott Real time PCR (Abbot, USA). HIV-1 Amplification Strategy with a detection limit of 40 copies/mL. The hemoglobin and the fasting blood sugar levels were determined in the third tube using a standard method and the « ULTRA ONE TOUCH” glucometer (LifeScan, Inc.,USA) respectively. The level of glycemia higher than ≥ 110 mg/dL was identified like cardiovascular risk factors.

### Statistical analysis

Data were presented as mean ± standard deviations or when indicated as percentage. Chi-square was performed to determine the significance of differences in the prevalence of opportunistic infections in both groups. Student’s *t*-test version 3.3.2 was used to compare the CD4 lymphocytes counts and viral load of both groups. The statistical SPSS program (version 16.0) was used for all the analysis. Results were considered significant at *p*-value less than 0.05.

## Results

A total of 169 patients were enrolled on the study among them 50 men and 119 female (Fig. 1). The women were more represented with a sex ratio of 2.38. After 6 months 27 patients (17.53 %) were dropped from the study these included 4 subjects (2.59 %) from the control group and 23 subjects (14.93 %) from spirulina group. These patients were dropped out because there were HIV–negative due to the error of screening test (6.89 % (6/87) in the control group and 3.65 % (3/82) in the spirulina group) or due to the reduction of CD4 cells count lower than 350 cells/mL (2.29 % (2/87) in the control group and 19.5 % (17/82) in the spirulina group) (Fig. 1).

Clinical characteristics of the 169 HIV–infected antiretroviral naïve patients are presented in (Table 1). The results clinical characteristics of patients in the Spirulina group and control showed at the beginning of the study, the presence of sexual transmitted infections (3 %, 16 %), malaria (6 %, 13 %) and Zona (3 %, 7 %). After 12 months most of these clinical manifestations significantly reduced in the group of patient who added spirulina to their diet. As for lifestyle, the percentage of patients who practiced physical activity (50 %, 60 %), drank alcohol and smoke tobacco respectively (3; 20 %) (1 %; 4 %) did not change.

**Table 1:** Clinical characteristics parameters of patients of the study

Parameters	T0				T6				T12			
	Spi	(%)	Ctrl	(%)	Spi	(%)	Ctrl	(%)	Spi	(%)	Ctrl	(%)
Malaria	8	9.1	2	2.29	5	4.74	21	62.5	03	37.5	18	20.68
Stomach ache	12	13.7	18	20.68	7	8.04	18	20.68	5	5.74	13	14.94
Chronic fatigue	24	27.4	11	12.64	4	4.56	8	9.2	2	2.29	12	13.1
Lack of appetite	11	12.64	17	19.54	8	9.1	6	6.89	1	1.14	8	9.2
More of appetite	0	0	0	0	22	25.28	14	16.09	23	26.43	9	10.34
Diarrhoea	0	0	0	0	0	0	8	9.1	0	0	0	0
Vomits	0	0	1	0	1	1.14	4	4.56	1	1.14	1	1.14
Vaginal itches	4	4.59	5	4.74	3	3.44	2	2.29	3	3.44	3	3.44
Candidiasis	5	5.74	4	4.56	0	0	4	4.56	0	0	3	3.44
Cough	0	0	0	0	2	2.29	6	6.89	1	1.14	8	9.1
Pinples on the skind	2	2.29	2	2.29	3	3.44	1	1.14	3	3.44	3	3.44
Others sikness	2	2.29	4	4.67	3	3.44	3	3.44	3	3.44	3	3.44
Alcohol	4	4.56	6	6.89	3	3.44	4	4.56	3	3.44	4	4.56
Smoking	2	2.29	3	3.44	2	2.29	3	3.44	1	1.14	2	2.29

*Ctrl* Control, *Spi* Spirulina group; T0, T6, T12: time (months)

The results presented the demography of the population of study showed that there were more women (76.1 %) than men (23.9 %) in the study with a sex ration of 2.38 (Table 2). The two groups (control and spirulina) were similar with respect to age, sex. The mean age was 35.6 ± 9 years. The value of the BMI was no significantly differences during the twelve months in both groups. The fasting blood sugar was decreased significantly after 12 months in the group of patients who added 10 g of *S. platensis* in their diet compare to the control group. No significant difference was observed in fasting blood sugar in both groups at the first 6 six months. The level of Hb was significantly higher in the spirulina group after 6 months compared to the control group. These results demonstrated that *S. platensis* could ameliorate the fasting blood sugar and the hemoglobin of the HIV-naïve patients.

**Table 2:** Demographic and clinical characteristics of patients during the trial

Variables	Ctrl/Spi	Period	Patients received local balance diet	Patients received spirulina combined with a local balance diet	*p*-value
Age (Years)	87/82	T0	35.43 ± 10.04	36.01 ± 9.44	0.58
Sex (%)					
Female	87/82	T0	64.5 % (56)	69.8 % (60)	0.06
Male			35.6 % (31)	25.6 % (22)	
BMI (kg/m^2^)	87/82	T0	26.02 ± 4.97	25.29 ± 4.54	0 .33
	79/80	T6	25.18 ± 6.41	25.41 ± 4.98	0.80
	66 /79	T12	22.18 ± 1.07	23.26 ± 6.53	0.44
CD4 (cells/μL)	87/82	T0	569.40 ± 179.89	596.32 ± 198	0.36
	79/80	T6	464.86 ± 200.33	609.07 ± 149.14	<0.00*
	66/79	T12	429.04 ± 177.19	614.92 ± 179.43	<0.00*
Viral load (copies/mL)	87/82	T0	61199.03 ± 6.27	74770.33 ± 3.19	0.53
	79/80	T6	66615,45 ± 7.45	38539.70 ± 2.22	<0.00
	66/79	T12	174595.22 ± 1.20	30872.33 ± 3.93	<0.00
Hemoglobin (mg/dL)	87/82	T0	12.87 ± 1.53	13.14 ± 1.85	0.32
	79/80	T6	12.22 ± 1.42	13.49 ± 1.24	<0.00
	66/79	T12	11.69 ± 1.62	12.71 ± 1.71	<0.00
Fasting Blood Sugar (mg/L)	87/82	T0	115 ± 53.70	105.89 ± 25.91	0.159
	79/80	T6	105.00 ± 30.76	103.14 ± 15.93	0.65
	66 /79	T12	113.77 ± 61.85	95.35 ± 9.63	<0.00*

Mean values ± SD. *Significant difference between the group (*P* < 0.00). Significant difference between the group (*p* < 0.05). Ctrl: Control, Spi: Spirulina group; To, T6,T13: time (months)

### Effects of *S. platensis* on the CD4 lymphocytes T and viral load

**Fig. 2:**
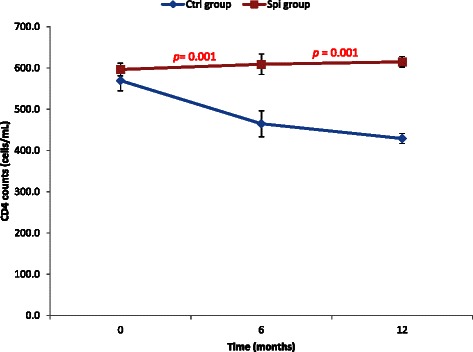
Variation of CD4 cells count during the period of experiment. Values are expressed as mean values ± SD, Ctrl: Control, Spi: Spirulina group; n = 78 after 6 months and n = 68 after 12 months; p < 0.01

**Fig. 3:**
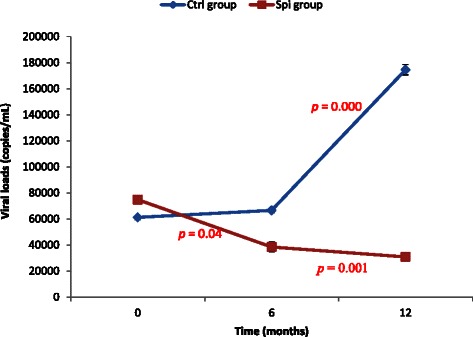
Variation of viral load of the groups during the experiment. Values are expressed as mean values ± SD, Ctrl: Control, Spi: Spirulina group; n = 78 after 6 months and n = 68 after 12 months; *p* < 0.01

To investigate the effects of *S. platensis* on the immune response of the HIV-naives patients, the CD4 cells count and the viral load of the patients were determined. The results of the CD4 cells count showed that at the baseline of the study there was no significant variation between patients of control and spirulina group. After 6 months of intake of spirulina powder these values linearly (R^2^ = 0.95) and significantly increased until 12 months in the spirulina group while in the control group a linear reduction (R^2^ = 0.96) of CD4 cells was noted (Fig. 2). In contrary, the viral load of the spirulina group significantly reduced while that of the control group increase significantly during the experiment (Fig. 3). These results showed that spirulina supplementation intake positively and significantly stimulated the immune system and inhibit the replication of viral on HIV-l naïve patients.

## Discussion

This present randomized control study included 169 patients divided into two groups either 87 (control group) or 82 (spirulina group). Our sample size and the duration of the experiment were respectively higher and longer than previous study conducted in Cameroon which had 73 and 53 patients for 12 weeks [14, 18]. The choice of a balance local diet for the control group was due to the fact that, the resources setting were limited and this approach could reduce nutritional deficiency and improve the status of the HIV treatment naïve patients. In addition, adequate nutritional status supports immunity and physical performance [6, 11]. This result also corroborates studies which report that adequate nutrition promotes and maintains optimal immune function [19]. We demonstrated in this study that supplementation with *S. platensis* during 06 months and after 06 months of follow up resulted in an increase CD4 count and hemoglobin level, a decrease of viral load. The improvement of the well-being of the patients through the regression of opportunistic diseases was also found in this group compared to the control group. This result confirms that individuals receiving *S. platensis* were significantly more likely to schedule clinic visits than those not receiving food assistance [9]. Several investigators have shown that adequate nutrition associated with or without education and counseling can improve nutritional status both during stable treatment free period and during severe episodes of the infection [20]. Our results showed that more than 50 % of patients from spirulina group found the significant regressions of opportunistic disease. This result is due to the presence of a large amount of macro and micronutrient identified in *S. plantensis* which can improve the immune system or prevent these opportunistic diseases [2, 21]. Several studies confirmed the beneficial effects of *S. platenis* supplements, vitamins or minerals on the immune system of VIH patients [14, 22]. Our results showed that, after 06 months of *S. platensis* supplementation intake, the patients of the intervention group continued to show a significant decrease of the viral load and an increase of the CD4 count compared to the control group (Fig. 3). Feeding studies showed that even in small amounts it can build up both humoral, cellular arms of the immune system and also enhances the body’s ability to generate new blood cells [12, 23]. Extract of *S. platensis* has been reported to possess *in vitro* antiviral and anti-AIDS activities [12, 24]. Also several *in vitro* and *in vivo* antiviral mechanisms of *S. platensis* extract have suggested that (*i*) the *S. platensis* extract acts by avoiding the virus to attach and penetrate to the cell membrane to infect the cell [24]. Therefore, the stuck virus cannot replicate and should be eliminate by natural defense of the body; (*ii*) the calcium-spirulant (Ca-SP) and sodium spirulant (Na-SP) found in the *S. platensis* extract interfered with replication of viruses including HIV-1, they increase the activation of macrophages, T-and NK cell activities [12, 25, 26]; (*iii*) Polysaccharides, phycocyanin, glycolipids and sulfolipids idenfied the *S. platensis* extract increase the immunity by enhancing bone marrow reproduction, growth of thymus and spleen and biosynthesis of serum protein; (*iv*) the presence of phenolic acids, tocopherols and ß-carotene and other antioxidant molecules identified in *S. platensis* exhibited the antioxidant protection with many benefits both *in vitro* and *in vivo.* Since the baseline characteristics between the two groups were similar, the beneficial effects noted in the Spirulina group demonstrated that nutritional supplementation for 6 months led to an improved recovery of the immune system and an improvement in the body’s ability to fight infections.

## Conclusion

This clinical trial demonstrated that six months supplementation of *S. platensis* and six months of follow up of HIV-infected, ART-naïve patients in the early stages of disease significantly delayed time to HIV disease progression and reduced the opportunistic diseases. Daily consumption of *S. platensis* coupled with nutritional counseling significantly was found to be strongly associated to the significantly reduction of the viral load and increase the CD4 cells counts but the mechanism by which *S. platensis* may induce these beneficial effects is not well understood. Other studies need to be conducted on the HIV-naive patients to elucidate this mechanism.
